# Comparison between conventional-dose and high-dose rocuronium use in general anesthesia for Cesarean section

**DOI:** 10.7150/ijms.95061

**Published:** 2024-04-08

**Authors:** Boo-young Hwang, Dowon Lee, Saeromi Chung, Hyundoo Hwang, Seung Chul Kim, Jae-young Kwon

**Affiliations:** 1 Department of Anesthesia and Pain Medicine, Biomedical Research Institute, Pusan National University Hospital, Busan, Republic of Korea.; 2 Department of Anesthesia and Pain Medicine, School of Medicine, Pusan National University, Busan, Republic of Korea.; 3 Department of Electrical and Computer Engineering, University of California, San Diego (UCSD), USA.; 4 Bredis Healthcare Inc., Kwangju, Republic of Korea.; 5 Department of Obstetrics and Gynecology, School of Medicine, Pusan National University, Busan, Republic of Korea.

**Keywords:** Anesthesia, General, Anesthesia, Obstetrical, Cesarean Section, Rocuronium

## Abstract

**Background:** There have been few studies comparing the effects of high- and low-dose rocuronium during cesarean section by directly measuring the concentration. Therefore, we conducted a study to examine the blood concentrations and clinical effects of both doses of rocuronium on mothers and fetuses.

**Methods:** Eighteen patients were randomly assigned to two groups: C Group (0.6 mg/kg), and H Group, (1.0 mg/kg). The primary outcome was the comparison of umbilical vein rocuronium concentration between two groups. We assessed ease of intubation, time from rocuronium administration to some TOF points, post-anesthesia care unit (PACU) stay time, infused remifentanil dose, maternal rocuronium concentration, and Apgar scores.

**Results:** No differences were observed in demographic data, ease of intubation, PACU stay time, 1 min Apgar scores, umbilical venous blood gas analysis between both groups. However, the time from rocuronium administration to T3 disappearance was shorter (*p*=0.009) and time to T1 and T2 reappearance were longer (*p*=0.003,* p*=0.009) in H group than that in C group. The administered remifentanil dose (*p*=0.042) was lower in the H group than in the C group. Rocuronium concentrations in the umbilical vein (*p*=0.004) and maternal vein before cord clamping (*p*=0.002) and at discharge (*p*<0.001) were also found to be higher in the H group than in the C group.

**Conclusions:** We observed no prolongation of PACU stay, and no differences in Apgar scores in H group compared to C group. It suggests that 1.0 mg/kg of rocuronium has no negative effects on the fetus and mother in cesarean section.

## Introduction

Although the use of regional anesthesia has led to a decrease in anesthesia-related maternal mortality during cesarean section, general anesthesia remains necessary in certain cases. These cases may include obstetric indications (e.g., postpartum hemorrhage), maternal indications (e.g., patient's refusal to receive neuraxial anesthesia), or contraindications to neuraxial anesthesia (e.g., anticoagulant use or coagulopathy) 1. However, general anesthesia poses a challenge in the airway management of obstetric patients because of physiological changes during pregnancy, which can result in severe complications 2.

Muscle relaxants are commonly used to facilitate tracheal intubation and to improve surgical conditions. Rocuronium is a muscle relaxant commonly used worldwide, and there have been several studies on its pharmacological and pharmacokinetic actions 3, 4. It provides good surgical conditions for fetal delivery and rapid sequence intubation. Although the standard dose for general anesthesia is 0.6 mg/kg, a higher dose of 1.0 mg/kg is used for rapid induction 5, 6. In our previous study, we developed a reliable electrochemical sensor capable of rapidly measuring rocuronium concentration 7.

In this study, we investigated the effects of conventional and relatively high doses of rocuronium on the mother and fetus during cesarean section by directly measuring the blood concentration using an electrochemical rocuronium sensor.

## Materials and methods

### Study design

We conducted a prospective, randomized, double-blind study at the Department of Anesthesia and Pain Medicine at Pusan National University Hospital in Korea from June 2018 to March 2020. The Institutional Review Board for Human Experiments approved this study, and it was registered with the Clinical Research Information Service, which conforms to the World Health Organization International Clinical Trials Registry Platform (WHO-ICTRP), under the registration number KCT0004986. Informed consent was obtained from all participants, in accordance with the principles of the Declaration of Helsinki, 2013.

### Participants

Eighteen patients, classified as American Society of Anesthesiologists physical status (ASA) classification I and II, and scheduled to undergo a cesarean section, were randomly assigned to one of two groups: the C group, which received a conventional dose of rocuronium (0.6 mg/kg), and the H group, which received a higher dose of rocuronium (1.0 mg/kg). Randomization was performed using a computer-generated random number list with block randomization. Patients who were not eligible for neuromuscular blocker treatment, those with a history of myopathy, psychological disorders, renal or hepatic failure, or preoperative administration of neuromuscular blockers were excluded.

### Treatment

After the patients arrived in the operating room, they were positioned with a leftward displacement of 15°. Routine perioperative monitoring devices were then attached, and baseline vital signs were checked. The depth of anesthesia was measured using the bispectral index (BIS, XP version 4.1; Aspect Medical Systems, Newton, MA, USA) monitoring. For monitoring the depth of neuromuscular relaxation, a 40-mA Train of four (TOF) stimulation (four pulses of 0.2ms in duration, at a frequency of 2 Hz, 2s in duration, TOF-watch, Organon S, Netherlands) was used to monitor the depth of neuromuscular relaxation.

Induction was performed using thiopental (4 mg/kg). After loss of consciousness, rocuronium was administered according to the group dose (C or H) by a nurse blinded to the patient group. Anesthesia was maintained with desflurane, 50% nitrous oxide, and oxygen. Following the operation, patients received pain control via a patient-controlled analgesia (PCA) device (GemStar® Infusion System, Hospira, IL, USA). PCA was initiated at the end of surgery, and the bolus dose was 1 ml (oxycodone 1 mg and nefopam 1 mg), with a lockout interval of 6 min and a 4-h limit of 40 ml. After the operation, 10 mg of pyridostigmine and 0.4 mg glycopyrrolate were administered to all patients, who were then transferred to the post-anesthesia care unit (PACU) and remained there until their Aldrete scores were > 8. If TOF was less than 0.2 after the surgery, sugammadex (2 mg/kg) was administered.

### Assessment

The main objective of our study was to compare the concentration of rocuronium in the umbilical cord vein between these two groups. We also measured the concentration of rocuronium in the maternal vein, the ease of intubation, which was rated on a scale of 0 to 9 based on the degree of jaw relaxation, vocal cords, responses during intubation: jaw was impossible to open, vocal cords were closed, and severe coughing or bucking = 0, jaw opens with difficulty, vocal cords were closing, and mild coughing = 1, jaw relaxation was moderate, vocal cords were moving, and slight diaphragmatic response to intubation = 2, jaw relaxation was fully done, vocal cords were relaxed, and no movement to intubation = 3. Additionally, we recorded the time from rocuronium administration to T3 disappearance, cord clamping, and reappearance of T1 and T2, TOF>0.9, eye opening. Other measures included PACU stay time, total concentration of infused remifentanil, and Apgar scores at 1 and 5 min after birth. Pediatric doctors assessed the Apgar score.

Blood samples were obtained from the maternal vein immediately before cord clamping and after the Aldrete score reached 9. Umbilical venous blood samples were collected immediately after cord clamping.

### Electrochemical experiments

We used a rocuronium measurement electrode developed in a previous study 7. In prior research, we developed an electrode and amperometic analysis method with accuracy and speed, and we verified that the concentration measured was accurate, through various electrochemical measurement methods. The manufactured electrode was stored in a refrigerator after preliminary preparation. The prepared electrodes were stored in 4 ºC refrigerator until use.

Prior to the assay, 60 µl of 0.1 M PBS was loaded on the electrodes and they were stabilized by cycling potential from 0.4 to 0.8 V (three cycles). To begin the assay, the 60 µl of blood samples were introduced onto the electrochemical sensors and incubated for 3 min. Finally, chronoamperometry was recorded at 0.65 V and data points were obtained at 5 s.

Fourteen maternal and fetal blood samples were collected in EDTA acid-treated anticoagulant blood vacuum tubes. Fresh blood samples were collected from volunteers and used for the assay. To quantify the rocuronium concentration, we analyzed known amounts of rocuronium samples (control solutions of 0, 1, 3, 5 µg/ml) using chronoamperometry at an applied potential of 0.65V (vs. Ag/AgCl). The change in the current (ΔI) was measured for each sample from the baseline signal at 5-s and a calibration plot was obtained. Using a calibration plot, we calculated the rocuronium concentration in the patient's blood.

### Statistical analysis

Data were analyzed using independent *t* test or the Mann-Whitney U test and chi-square test. Continuous data are presented as mean ± standard deviation or median ± interquartile range, and categorical data are presented as numbers (percentages). Statistical significance was set at *p* < 0.05. All statistical analyses were conducted using the Statistical Package for the Social Sciences software (SPSS v. 21.0; IBM Inc., Chicago, IL, USA).

## Results

No demographic differences were observed between the two groups. Additionally, no significant differences were found in intubation ease, time from rocuronium administration to skin incision, cord clamping, TOF ratio > 0.9, eye opening, extubation, PACU stay time, 1 min and 5 min Apgar score and venous blood gas analysis of the blood from the umbilical vein between both groups. However, the time from rocuronium administration to T3 disappearance was shorter in H group than in C group (Table 2, *p*=0.009). The time from rocuronium administration to T1 and T2 reappearance were longer in H group than in C group (Table 2, *p*=0.003, 0.009). The administered remifentanil dose was lower in H group (Table 1, *p*=0.042), and the rocuronium concentrations in both maternal vein immediately before cord clamping and after the Aldrete score reached 9 and umbilical vein immediately after cord clamping were higher in H group than in C group (Table 1, 3, *p*=0.004, 0.002, and <0.001, respectively).

## Discussion

The amount of muscle relaxant needed varies from patient to patient owing to differences in surgeon preference, operation duration, and type of surgery. In a cesarean section, adequate muscle relaxation must be achieved to facilitate difficult airway manipulation, rapid sequence induction, and delivery of the baby through an incision.

The usual induction dose for rocuronium is 0.6 mg/kg (2 × ED95 of adductor pollicis), which is considered safe and effective for cesarean section 5. However, conventional doses of rocuronium have been found to result in inferior intubation conditions and delayed onset of muscle relaxation compared to succinylcholine 8. In our study, both groups had a similar ease of intubation; however, the time from administration of rocuronium to T3 disappearance was shorter in C group than in H group. As we waited until T3 disappeared, there was no difference in movement or jaw relaxation during intubation between the two groups.

We administered a high dose of rocuronium (2 x ED_95_ to the laryngeal muscle and diaphragm), which is known to cause rapid sequence induction 9, 10. One common side effect of neuromuscular blockers is residual neuromuscular blockade, which is defined as a TOF ratio of less than 0.7 at the adductor pollicis. However, this threshold was recently increased to 0.9 11. Rocuronium is primarily metabolized in the liver, with variable elimination 12 through the kidney (12-33%) 4. Therefore, kidney and liver impairments can affect the pharmacology of rocuronium. Some studies have shown that the use of only one intubation dose can lead to residual postoperative paralysis 13. In one case, an extremely prolonged neuromuscular blockade (>10 h) occurred following a 0.9 mg/kg dose of rocuronium 14. Women appear to be more sensitive to a single dose of rocuronium than men 15, and the neuromuscular blockade induced by rocuronium can last longer in pregnant women than in non-pregnant women 16. In our case, the rocuronium concentration in the H group was higher than that in the C group at discharge, but we observed no prolongation of PACU stay. Based on our findings, we concluded that 1.0 mg/kg of rocuronium could be safely used for cesarean section operations without prolonging PACU stay or delaying recovery.

A few studies have investigated the placental transfer of rocuronium. Placental transfer depends on the molecular weight, lipid solubility, polarity, pKa, and protein binding. Muscle relaxants are highly ionized, which reduces placental transfer and results in minimal effects on fetal and uterine smooth muscles 2. Rocuronium bromide has a molecular weight of 609.7 D and is approximately 30% bound to human plasma proteins. Therefore, they undergo incomplete transfer across the placenta 17. The placental transfer of vecuronium was 0.11, and pancuronium was 0.19, as indicated by the umbilical vein/maternal vein ratio 18. Wierda revealed that only limited placental transfer occurred with rocuronium and suggested that administration of an intubating dose did not cause paralysis or hamper the vital functions of the newborn 19. Abouleish et al. also showed that there were no adverse effects of rocuronium on neonates, as evaluated by 1- and 5-min Apgar scores, time to sustained respiration, total and muscular neuroadaptive capacity scores, acid-base status, and blood-gas tensions in umbilical arterial and venous blood 8. However, a high rocuronium (1.0 mg/kg) was associated with lower Apgar scores at 1 min 20. In our study, there was no difference in the Apgar score despite the difference in rocuronium concentration in umbilical vein. These findings suggest that rocuronium has minimal effects on the fetus, although a portion of rocuronium passes across the placenta and into the umbilical vein. The ratio of plasma rocuronium concentration in the umbilical vein to that in the maternal vein (UV/MV) was 0.161 in a previous study 8 but 0.175 in our study. Previous studies have demonstrated an increase in UV/MV with a longer time from drug injection to delivery for drugs with a high molecular weight, such as atracurium; however, conflicting results have been reported for pancuronium. This effect has not been observed for drugs with low molecular weights, such as vecuronium 21. Therefore, it may be advantageous to shorten the interval between rocuronium injection and delivery to prevent an increase in the UV/MV ratio.

Electrochemical biosensors have advantages such as simple operation, low cost, robustness, high sensitivity, easy miniaturization, small sample volumes, and the ability to be used in biofluids. They are powerful tools widely used in medical, industrial, and environmental applications to enable POCT. In a previous study, we developed a rocuronium sensor by measuring its oxidation current, which showed a proportional response to its concentration 7. We demonstrated the sensor's ability to detect rocuronium in a previous study (0.025 to 10 μg/mL dynamic range, with a detection limit of 3.83 ng/mL), which also showed applicability to blood samples within 4 min.

In this study, we did not measure the plasma concentration of 17-desacetylrocuronium, which has a weak neuromuscular blocking effect. To accurately assess the effect of muscle relaxants on both the fetus and mother, it would be beneficial to measure metabolites using this method.

Our study revealed that the concentration of rocuronium in the maternal vein as well as in the umbilical vein were higher in H group than in C group. Although there were no differences in patients' recovery or Apgar scores between both groups, it was confirmed that the rocuronium was delivered to the umbilical vein in proportion to the concentration of administered rocuronium, so it cannot be certain that there will be no negative effects on the fetus or mother when rocuronium concentrations higher than 1.0 mg/kg are used. Therefore, further research is needed to identify the appropriate concentration of rocuronium that can provide effective anesthesia while minimizing its effects on the fetus and mother.

## Figures and Tables

**Table 1:** Maternal characteristics

		Group C (n=9)	Group H (n=9)	*p*-value
ASA classification, n (I/II)		4/5	4/5	0.681
Mallampati score, n (%)	1	5 (55.6)	3 (33.3)	0.077
	2	4 (44.4)	5 (55.6)	
	3	0	1 (11.1)	
Easiness of intubation, n (%)	9	7 (77.8)	6 (66.7)	0.783
	8	1 (11.1)	1 (11.1)	
	7	1 (11.1)	1 (11.1)	
	6	0	1 (11.1)	
Age (years)		32.3 ± 3.4	34.4 ± 2.9	0.181
Height (cm)		163.9 ± 4.4	162.4 ± 5.8	0.536
Weight (kg)		73.2 ± 9.1	73.6 ± 7.1	0.825
Gestational age (weeks)		37.8 ± 0.8	37.9 ± 0.3	0.903
Duration of anesthesia (min)		94.1 ± 15.4	89.2 ± 12.3	0.395
Duration of surgery (min)		76.3 ±13.3	69.9 ± 9.5	0.468
Remifentanil consumption (  g)		274.4 ± 151.9	141.1 ± 83.6	0.042*
PACU stay (min)		18.8 ± 5.7	23.0 ± 5.8	0.199
Concentration of rocuronium in maternal vein before cord clamping (  g/mL)		0.193 ± 0.1	0.344 ± 0.9	0.004*
Concentration of rocuronium in maternal vein at discharge from PACU (  g/mL)		0.015 ± 0.006	0.027 ± 0.004	0.000^ϯ^

Data are presented as mean ± standard deviation or number (percentage). ASA classification= American Society of Anesthesiologists physical status (ASA) classification. PACU= post-anesthesia care unit. Ease of intubation is rated on a scale of 0 to 9 based on the degree of jaw relaxation, vocal cords, responses during intubation: jaw was impossible to open, vocal cords were closed, and severe coughing or bucking = 0, jaw opens with difficulty, vocal cords were closing, and mild coughing = 1, jaw relaxation was moderate, vocal cords were moving, and slight diaphragmatic response to intubation = 2, jaw relaxation was fully done, vocal cords were relaxed, and no movement to intubation = 3. Group C: Conventional concentration of rocuronium (0.6 mg/kg), Group H: High concentration of rocuronium (1.0 mg/kg). The statistical significance level of *p* < 0.05 was denoted by *, and *p* < 0.01 was denoted by ^ϯ^. There were no significant differences observed between the groups in terms of ASA classification, Mallampati score, ease of intubation, age, height, weight, gestational age, duration of anesthesia, duration of surgery, and PACU stay. However, the concentration of rocuronium in the maternal vein before cord clamping and at discharge from PACU was lower in Group C than in Group H. The administered remifentanil concentration was higher in Group C than in Group H.

**Table 2:** Time from administration of rocuronium

	Group C (n=9)	Group H (n=9)	*p*-value
T3 disappearance (min)	2.7 ± 0.5	1.8 ± 0.3	0.009*
Skin incision (min)	3.4 ± 1.0	2.9 ± 1.1	0.258
Cord clamping (min)	10.0 ± 3.1	8.3 ± 2.0	0.102
T1 reappearance (min)	26.4 ± 5.3	42.8 ± 9.7	0.003*
T2 reappearance (min)	37.5 ± 8.3	54.6 ± 13.3	0.009*
TOF ratio=0.9 (min)	74.7 ± 11.12	74.2 ± 9.6	0.965
Eye opening (min)	74.6 ± 12.7	71.5 ± 10.0	1.000

Data are presented as mean ± standard deviation. TOF ratio= Train of four ratio. Group C: Conventional concentration of rocuronium (0.6 mg/kg), Group H: High concentration of rocuronium (1.0 mg/kg). The statistical significance level of *p* < 0.01 was denoted by *. T3 disappearance time in Group H was shorter than that of Group C. T1 and T2 reappearance time in Group H was longer than that of Group C. There were no differences observed between the two groups in terms of skin incision, cord clamping, TOF ratio of 0.9, or eye opening from the administration of rocuronium.

**Table 3:** Apgar score and laboratory data in umbilical vein

	Group C (n=9)	Group H (n=9)	*p*-value
Apgar score at 1 min	7.4 ± 0.5	7 ± 0.8	0.353
Apgar score at 5 min	9 ± 0	8.7 ± 0.5	0.065
Concentration of rocuronium in umbilical vein (  g/mL)	0.034 ± 0.02	0.059 ± 0.01	0.002*
pH	7.3 ± 0.02	7.3 ± 0.02	1.000
pCO2	48.6 ± 3.5	47.6 ± 4.3	0.401
pO2	42.9 ± 15.9	4.4 ± 16.7	0.965
Hb	14.0 ± 0.8	14.0 ± 1.4	0.594
O_2_ Saturation	67.4 ± 14.9	68.7 ± 17.0	0.895
HCO_3_	24.9 ± 1.5	24.8 ± 1.6	0.965
Base excess	-1.3 ± 1.2	-1.2 ± 1.4	0.965
Alveolar O_2_	90.1 ± 3.6	89.6 ± 6.0	0.965
Aa ratio	0.5 ± 0.2	0.5 ± 0.2	0.889

Data are presented as mean ± standard deviation or median ± IQR. Group C: Conventional concentration of rocuronium (0.6 mg/kg), Group H: High concentration of rocuronium (1.0 mg/kg). The statistical significance level of *p* < 0.01 was denoted by *. There were no differences observed between the two groups in terms of Apgar scores, pH, pCO_2_, pO_2_, Hb, O_2_ saturation, HCO3, or base excess of the umbilical vein. However, the concentration of rocuronium in umbilical vein was higher in Group H than in Group C.
